# A mathematical model separates quantitatively the cytostatic and cytotoxic effects of a HER2 tyrosine kinase inhibitor

**DOI:** 10.1186/1742-4682-4-14

**Published:** 2007-04-03

**Authors:** Peter Hinow, Shizhen Emily Wang, Carlos L Arteaga, Glenn F Webb

**Affiliations:** 1 Department of Mathematics, Vanderbilt University, Nashville, TN 37240, USA; 2 Department of Cancer Biology, Vanderbilt University, Nashville, TN 37232, USA; 3 Department of Medicine, Vanderbilt University, Nashville, TN 37232, USA; 4 These authors contributed equally to this study

## Abstract

**Background:**

Oncogene signaling is known to deregulate cell proliferation resulting in uncontrolled growth and cellular transformation. Gene amplification and/or somatic mutations of the HER2/Neu (ErbB2) proto-oncogene occur in approximately 20% of breast cancers. A therapeutic strategy that has been used to block HER2 function is the small molecule tyrosine kinase inhibitor lapatinib. Using human mammary epithelial cells that overexpress HER2, we determined the anti-proliferative effect of lapatinib through measuring the total cell number and analyzing the cell cycle distribution. A mathematical model was used to interpret the experimental data.

**Results:**

The model suggests that lapatinib acts as expected by slowing the transition through G_1 _phase. However, the experimental data indicated a previously unreported late cytotoxic effect, which was incorporated into the model. Both effects depend on the dosage of the drug, which shows saturation kinetics.

**Conclusion:**

The model separates quantitatively the cytostatic and cytotoxic effects of lapatinib and may have implications for preclinical studies with other anti-oncogene therapies.

## Background

Molecule-targeted anti-cancer drugs have been developed as a result of our understanding of tumor cell and molecular biology. Compared to "traditional" cancer therapies, targeted drugs such as the tyrosine kinase inhibitors (TKIs) have higher specificity and relatively lower toxicity in selected patients with corresponding oncogene expression. Members of the type 1 receptor tyrosine kinase (RTK) family, which includes the epidermal growth factor receptor (EGFR), HER2 (ErbB2), HER3 and HER4 play a crucial role in growth and differentiation of both normal and malignant mammary epithelial cells [1,2]. Binding of receptor-specific ligands to the ectodomain of EGFR, HER3 and HER4 results in the formation of receptor dimers and hetero-oligomers to which HER2 is recruited as the preferred heterodimerization partner [3]. HER2 gene amplification has been reported in approximately 20% of breast cancers, where it is associated with poor patient outcome [4]. Studies with HER2-overexpressing breast cancer cell lines and human tumors have shown constitutive phosphorylation of HER2 [5,6]. Overexpression of HER2 is associated with transformation of mammary epithelial cells [7,8] as well as shorter survival in patients with breast carcinoma [4,9]. These facts make HER2 a rational therapeutic target in human breast cancer. One therapeutic approach against HER2-overexpressing breast cancers is the generation of trastuzumab, a humanized IgG1 that binds to residues 529–626 in domain IV of the HER2 ectodomain [2,10]. However, many patients with HER2-overexpressing advanced disease do not respond clinically to trastuzumab and many that initially respond eventually relapse with antibody-resistant disease. Lapatinib (GW572016, GlaxoSmithKline) is a selective reversible inhibitor of both EGFR and HER2 tyrosine kinases. Lapatinib mimics ATP and binds to the ATP site in the tyrosine kinase domain of HER2, resulting in blockade of the receptor's catalytic activity [11].

Preclinical data have shown that tumor cells overexpressing EGFR or HER2 are growth inhibited by lapatinib both in vitro and in vivo [12-14]. Lapatinib inhibits the activation of cell proliferation effectors, Erk1/2 (also known as mitogen-activated protein kinase, or MAPK) and AKT, which are downstream of EGFR and HER2 [11,15]. In another study in which over 30 breast cancer cell lines were tested for their responses to lapatinib, concentration-dependent antiproliferative effects of lapatinib were seen in all cells but varied significantly between individual cell lines [13]. Response to lapatinib is significantly correlated with HER2 expression and its ability to inhibit the phosphorylation of HER2 and downstream effectors. In phase II clinical trials, treatment with lapatinib resulted in objective tumor responses in 28% of patients with HER2-positive advanced breast cancer [12]. Modeling the antiproliferative effects of this oncogene inhibitor using mathematical tools will lead to novel insights into the functioning features and mechanisms of the inhibitor. The model may also provide constructive clinical implications, such as the predictive effects of the inhibitor in first-line therapy in combination with chemotherapy.

In this study we used MCF10A human mammary epithelial cells engineered to overexpress HER2 in order to determine the anti-tumor effects of lapatinib. Compared to control MCF10A cells that do not overexpress HER2, MCF10A/HER2 cells exhibit a gain-of-function phenotype including increased proliferation and filling of the lumen when grown in three dimensions, as a result of oncogene overexpression [16]. Lapatinib inhibits the phosphorylation and function of HER2 in these cells and suppresses growth [16]. At the molecular level the functional mechanisms of HER2 inhibitors are evaluated by the activities of downstream signaling networks, which are often determined by immunoblots. However, signaling pathways such as the PI3K/Akt and the MEK/Erk pathways can converge at various levels of the signaling cascades, making it difficult to separate a combined effect on cell growth and survival. Quantitative models can separate the strengths of drug action on individual phases of the cell cycle. Previous molecular biological studies have shown that HER2 is associated with increases of both G_1_-S-specific cyclins (cyclins D and E) and G_2_-M-specific cyclin (cyclin A) [17,18], which are crucial for G_1_-S and G_2_-M progression, respectively. Our objective in this study is to use quantitative models to determine if HER2 inhibitors abolish the function on both phase transitions and how this contributes to cell cycle blockage.

Mathematical modeling has been applied extensively to study the growth kinetics of tumors, with and without treatment; see [19-26] and the references therein. These authors have focused on phenomena such as decelerated growth, quiescence, homeostasis and chemotherapy scheduling.

It has been recognized that, apart from killing cells outright, anticancer drugs can also act by delaying the progression through the cell cycle. Moreover, this blocking effect can be phase specific [27]. Transition through one phase of the cell cycle may be delayed while transition through another phase is unaffected. Mathematical modeling here provides the tool to test possible alternative scenarios against each other and to gain new insight. In a series of papers, Ubezio and collaborators used a mathematical modeling approach to investigate phase-specific cytotoxic and cytostatic effects of drugs such as cisplatin, melphalan and topotecan *in vitro *[26-29]. A continuous model has been used by Agur and coworkers [22] to predict the effect of periodic treatments with cycle-specific cytotoxic drugs.

Our mathematical model consists of populations of proliferating and nonproliferating cells with individual cells distinguished by cell cycle position and is described in detail below. Numerical simulations of the model give good agreement with the experimental data. We find that the experimental data are consistent with a theory in which lapatinib preferentially affects cells growing in monolayer culture in G_1_-phase in a dose-specific manner. As the dose of lapatinib is increased, however, our study indicates that other phases of the cell cycle are affected as well. Moreover, we see a gradual onset of the cytostatic effect as opposed to a sudden onset. We observe a simple functional relationship between the strength of the cytostatic effect and the drug concentration (see equation (13) for details). Finally, our study indicates that a cytotoxic effect is present after longer periods of exposure to the drug.

## Results

**Figure 1:**
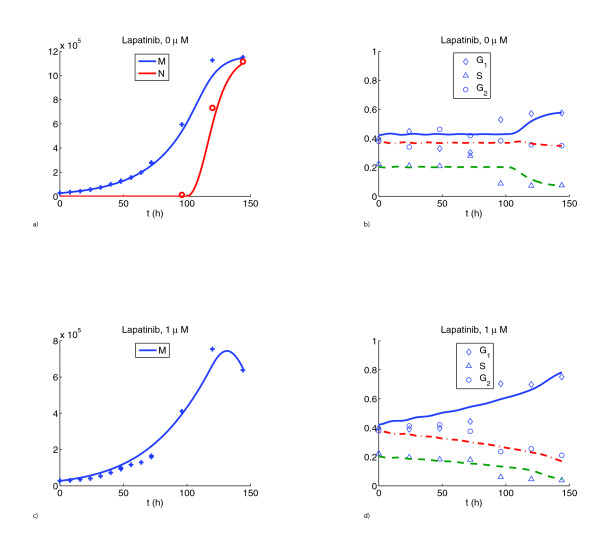
Total cell counts **A **and flow cytometric data **B **for untreated cells. Discrete symbols designate averages of three independent realizations of the experiment while curves are the predictions of our model. In panel **A **the total number of cells is shown in blue while the number of nonproliferating cells is shown in red. This prediction agrees well with the measurements in Figure 2 **B**. In panel **B **the fraction of cells in each phase is shown. In this and in the subsequent figures individual symbols mark experimental measurements while the curves are the corresponding simulations. Total cell counts **C **and flow cytometric data **D **for cells treated with 1 *μM *lapatinib. These graphs are representative of all drug treatment cases.

**Figure 2:**
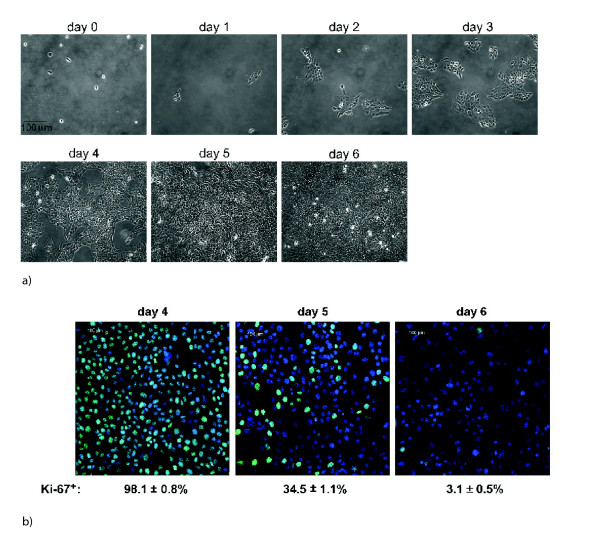
**A **Phase contrast images of untreated cells on different days. **B **Staining of untreated cells for marker of proliferation Ki-67 (green) on days 4 to 6. Blue: DAPI staining showing total nuclei. The simulations predict 100%, 40% and 4% proliferating fraction on days 4, 5 and 6, respectively (see Figure 1 **A**).

In the control scenario (without treatment) the cell counts showed an initial exponential increase of the population and then a leveling off (see Figure 1A). To explain this leveling off, the nonproliferating cell class was incorporated into the model. Nonlinear models with nonproliferating subpopulations have been used extensively to explain Gompertzian growth kinetics of tumors [20,21]. Proliferating cells enter the nonproliferating class irreversibly at a rate dependent on their maturity and the total population count of both proliferating and nonproliferating cells. This nonlinearity in the model accounts for the confluence observed in the control study on day 6. Staining of cells with the marker for proliferation Ki-67 showed a dramatic decrease of the proliferating fraction from day 4 to day 6 (see Figure 2B), well borne out by the numerical simulation. In the model, nonproliferating cells arrested their maturity value at the moment of transition from proliferation (see equations (2)–(3)). No mortality of cells was assumed in the model for the control, since no decrease in cell numbers was observed. In addition, staining for the marker of apoptosis Caspase-3 was negative for the control (data not shown). The flow cytometric data from the control scenario are shown in Figure 1B. Owing to uncertainty in the experimental measurements, there were some discrepancies in the fit, particularly during days 1 – 3.

**Figure 3:**
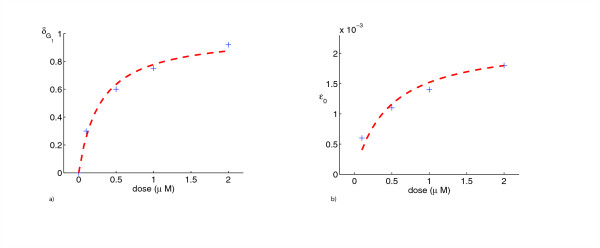
The values δG1
 MathType@MTEF@5@5@+=feaafiart1ev1aaatCvAUfKttLearuWrP9MDH5MBPbIqV92AaeXatLxBI9gBaebbnrfifHhDYfgasaacH8akY=wiFfYdH8Gipec8Eeeu0xXdbba9frFj0=OqFfea0dXdd9vqai=hGuQ8kuc9pgc9s8qqaq=dirpe0xb9q8qiLsFr0=vr0=vr0dc8meaabaqaciaacaGaaeqabaqabeGadaaakeaaiiGacqWF0oazdaWgaaWcbaGaem4raC0aaSbaaWqaaiabigdaXaqabaaaleqaaaaa@30C3@ (*d*) **A **and *ε*_0_(*d*) **B **as functions of dose show saturation (see equations (13)-(14) and Table 2)

**Figure 4:**
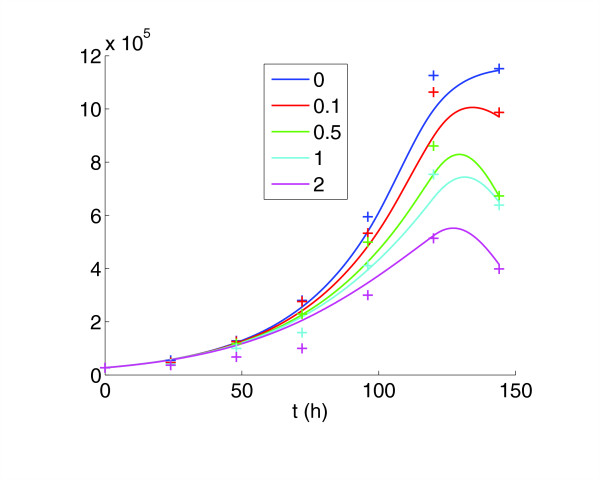
Combined cell counts and simulations for the control and various drug concentrations. The goodness-of-fit is comparable across the different drug doses.

**Figure 5:**
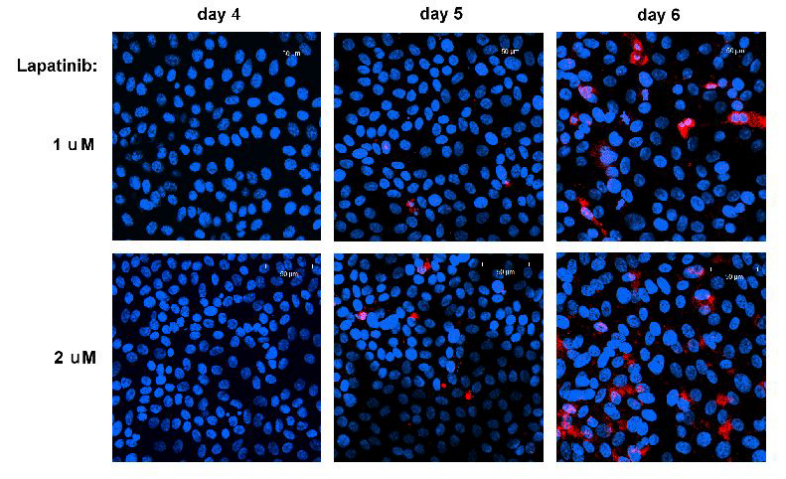
On days 4–6, cells treated with 1 and 2 *μM *lapatinib respectively are stained for cleaved Caspase-3 (red), a marker of apoptosis. Blue: DAPI staining showing total nuclei.

The model for the control case was used as a reference for the treatment cases, with two separate effects of the drug added. The first was the cytostatic effect, which slowed maturation velocity. Our numerical simulations indicate that lapatinib preferentially blocks cells in G_1_-phase. At higher dose (2 *μM*) the model also incorporates blocking effects in G_2_/M phase. We find that the strength of the cytostatic effect saturates at higher doses (see Table 1 and Figure 3A). The second effect of the drug was a cytotoxic action. This was incorporated into the model to explain the decrease in cell counts from day 5 to day 6, which was not present in the control (Figure 4). In the model it was assumed that this cytotoxic action only set in after 5 days. This is supported by experimental observations, as staining for the marker of apoptosis Caspase-3 was negative before day 5 (Figure 5). The model simulations agreed substantially with the experimental data, both in the total population counts and the flow cytometric data (Figure 1C, D).

**Table 1:** Numerical values of the parameters that are fixed throughout all scenarios

parameter	numerical value	remarks
aG1 MathType@MTEF@5@5@+=feaafiart1ev1aaatCvAUfKttLearuWrP9MDH5MBPbIqV92AaeXatLxBI9gBaebbnrfifHhDYfgasaacH8akY=wiFfYdH8Gipec8Eeeu0xXdbba9frFj0=OqFfea0dXdd9vqai=hGuQ8kuc9pgc9s8qqaq=dirpe0xb9q8qiLsFr0=vr0=vr0dc8meaabaqaciaacaGaaeqabaqabeGadaaakeaacqWGHbqydaWgaaWcbaGaem4raC0aaSbaaWqaaiabigdaXaqabaaaleqaaaaa@3062@	7 *h*	G_1_/S-boundary in absence of drugs
*a*_*S*_	11 *h*	S/G_2_-boundary in absence of drugs
*a*_*m*_	15 *h*	minimal age for division
*a*_*M*_	30 *h*	maximal cell age
*σ*	2	standard deviation of intermitotic times
*c*	0.22	nonproliferation constant
*M*_0_	6·10^5 ^cells	crowding threshold for nonproliferation
*μ*(*a*)	{1, 0.25, 0.6}	see Figure 6 **B**

## Discussion and conclusion

The mathematical model provided a means to separate the cytostatic and cytotoxic action of the drug in the experiments. We summarize our findings as follows.

### Cell cycle specificity of cytostatic effects

The strength of the cytostatic effects depends on the drug dosage; the drug showed saturation kinetics (see the last section and Figure 3A for details). At low concentrations (0.1 – 1 *μM*) only cells in G_1_-phase are delayed. At higher concentrations (2 *μM*) we hypothesize, on the basis of our model simulations, that cells in G_2_-phase are delayed as well and may be prevented from entering mitosis. It was not necessary to introduce a cytostatic effect for cells in S-phase. We therefore suggest that cells in S-phase remain unaffected at all concentrations.

### Dynamic behavior

Our numerical simulations indicate a buildup phase for the drug action that stretches over several days. Initially we assumed a sudden onset of the cytostatic action after a certain time. The simulations showed pronounced oscillations in the fractions of cells in each phase that were not observed in the data (simulations not shown). We then assumed a gradual increase (with respect to time) of the cytostatic effects. As the cycle time of an average cell is less than one day, we conjecture that the initial effects of the drugs alters a cell's protein contents but still allows division. Only after several generations is the progression of the cells through the respective cell cycle phases fully retarded. The same holds for the loss of cells. This can be explained by the functional mechanism of the drug and the nature of cell mitosis. Oncogene inhibitors such as lapatinib affect the activities of oncogene downstream effectors, which usually include regulatory proteins crucial for proliferation and survival. As cytoplasmic division occurs during mitosis, the inhibitory effect of lapatinib on these crucial effectors can be "inherited" as protein concentration in the cytosol, where the drug effect can further accumulate. Once the concentrations of these crucial proteins exceed certain thresholds, physiological effects such as growth arrest and apoptosis will be induced in the descendant cells. The conclusions that the length of lapatinib treatment is crucial for the overall drug effects, and that it takes several cell generations for the drug to show a clear cellular effect, especially at a lower concentration, may have potential clinical implications.

### The cytotoxic effect

A model with cytostatic action alone cannot lead to a decrease in total cell number, which made it necessary to introduce a cytotoxic effect. To explain the additional mortality of cells after 120 hours, we propose that cells in which progression through the cell cycle has been retarded for too long become prone to apoptosis in the presence of lapatinib. We conjecture that the strength of the cytotoxic effect also saturates at higher doses, as is suggested by Figure 3B.

While it is difficult to extrapolate conclusions from our *in vitro *study to the *in vivo *situation, the following suggestions are plausible. Lapatinib acts chiefly through slowing the progression of proliferating cells in monolayer culture. Furthermore, it is advisable to combine lapatinib with cytotoxic therapeutic agents that kill not only proliferating cells but also quiescent cells, such as some alkylating agents. These drugs may complement the antitumor effect of lapatinib and therefore serve as good candidates to be tested in combined treatment in the future.

We have shown that a mathematical model based on population dynamics can be applied to interpret the cytostatic and cytotoxic effects of lapatinib. Earlier mathematical models [27] used a discrete partition of the cell cycle into age compartments and also a discrete time scale. We find that continuum models are advantageous from the viewpoint of parametrization and computability. In particular, the number of free parameters in our model is significantly smaller than in previously proposed models and each has a straightforward biological interpretation. Both the cytostatic and cytotoxic effects of lapatinib are currently being investigated in various other HER2-overexpressing breast cancer cell lines. Our model can certainly be applied to other oncogene inhibitors that have cytostatic effects on cells during a specific phase of the cell cycle.

## Materials and methods

MCF10A/HER2 cells were generated and grown as described [30]. Lapatinib was kindly provided by Tona Gilmer (GlaxoSmithKline, Research Triangle Park, NC). Equal numbers of cells (3·10^4^/well) were seeded on 6-well plates for growth assay and cell cycle analysis over a time course of 6 days. At 4 *h *post seeding when > 98% of cells were attached to the plates, the cells were either harvested by trypsinization as day 0 samples, or treated by different doses (0.1, 0.5, 1 and 2 *μM*) of lapatinib, which was added and left in the medium throughout. Media containing fresh drug were replenished every 2 days. Cells were harvested by trypsinization every 8 *h *after adding drug for the initial 3 days and every 24 *h *for days 4–6. One tenth of the cells collected (100 *μl *out of 1 *ml*) were subjected to total cell number counting using a Coulter counter. The rest of the cells (900 *μl *out of 1 *ml*) were subjected to cell cycle analysis. For flow cytometric analysis of cell cycle distribution, cells were fixed in 70% ethanol for 24 *h *at – 20°C, and rehydrated in cold phosphate buffered saline (PBS) for 30 *min *on ice. Redydrated cells were subsequently labeled with 50 *μg/ml *propidium iodide (Sigma, St. Louis, MO) containing 125 units/*ml *protease-free RNase (Calbiochem, San Diego, CA) in the dark for 30 *min *at room temperature and filtered through a 95 *μm *pore size nylon mesh (Small Parts, Miami Lakes, FL). A total of 10,000 stained nuclei was analyzed in a FACS/Calibur Flow Cytometer (BD Biosciences, Franklin Lakes, NJ). All treatments were done in triplicate.

Indirect immunofluorescence assays (IFA) were performed as described previously [16] to detect markers for proliferation and apoptosis. Fluorescent images were captured using a Zeiss inverted LSM510 confocal microscopy system. Primary antibodies include Ki-67 (Calbiochem) and cleaved caspase-3 (Cell Signaling, Danvers, MA). The fluorescent antibodies were Oregon Green-*α*-mouse IgG and Texas Red-*α*-rabbit IgG (Molecular Probes, Carlsbad, CA).

**Figure 6:**
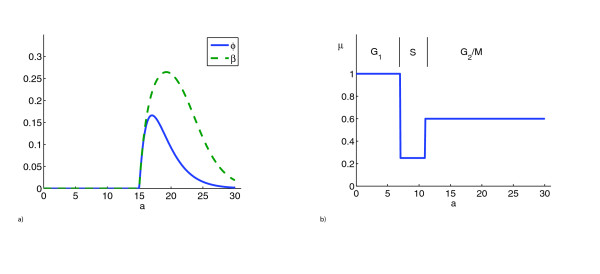
**A **The age-dependent probability of division *φ *(solid blue line) and the corresponding division rate *β *(dashed green line) are shown. *φ *is given by a shifted Γ-distribution with mean 19 *h *(equation (10)). Observe that the units of *a *are hours. However, cytostatic effects result in a slowed progression through the cell cycle. **B **We show the cell-phase-dependent tendency of cells to become nonproliferating *μ*(*a*) (see equation (7)).

The mathematical model was designed to quantify the cytostatic and cytotoxic effects of the drug on the basis of the population dynamics observed in the experiments. The model consists of a system of differential equations describing these dynamics over the 6 day time course (see the last section for a detailed description). Cells are classified as proliferating or nonproliferating. In the model, proliferating cells are tracked according to position in the cell cycle by assigning to each cell a variable called maturity. Maturity in the control scenario (0 *μM *lapatinib) corresponds to cell age (*i.e*., time that has passed since the last mitosis). The maturity values in the control delimiting the phases of the cell cycle are set at 0 – 7 *h *(G_1_), 7 – 11 *h *(S), and 11 – 30 *h *(G_2_/M) (see Figure 6B and the last section for further explanation). The model takes into account the variability of intermitotic times with mean age of division approximately 19 *h *in the control (see Figure 6A). The mathematical model was programmed using MATLAB (version 7.1, The MathWorks, Inc., Natick, MA). The codes are available upon request from the corresponding author. A standard upwind scheme is used for the numerical solution of the partial differential equations [31].

### The mathematical model

Let *t *≥ 0 denote the time since the beginning of the experiment and *a *∈ [0, *a*_*M *_] denote the maturity of a cell. This maturity variable can be thought of as the position of a cell in its cell cycle. We wish to emphasize that in the absence of cytostatic effects of drugs, maturity coincides with chronological age, the time since cell division. In experimental terms, maturity is measured by differential DNA-content. See the discussion in [26] for further information.

Let *p*(*a*, *t*) and *n*(*a*, *t*) denote the densities of proliferating and nonproliferating cells, respectively, of maturity *a *at time *t*. The total number of cells at time *t *is obtained by integrating both densities over the age space

M(t)=∫0aM(p(a,t)+n(a,t))da.
 MathType@MTEF@5@5@+=feaafiart1ev1aaatCvAUfKttLearuWrP9MDH5MBPbIqV92AaeXatLxBI9gBaebbnrfifHhDYfgasaacH8akY=wiFfYdH8Gipec8Eeeu0xXdbba9frFj0=OqFfea0dXdd9vqai=hGuQ8kuc9pgc9s8qqaq=dirpe0xb9q8qiLsFr0=vr0=vr0dc8meaabaqaciaacaGaaeqabaqabeGadaaakeaacqWGnbqtcqGGOaakcqWG0baDcqGGPaqkcqGH9aqpdaWdXaqaaiabcIcaOiabdchaWjabcIcaOiabdggaHjabcYcaSiabdsha0jabcMcaPiabgUcaRiabd6gaUjabcIcaOiabdggaHjabcYcaSiabdsha0jabcMcaPiabcMcaPiabbsgaKjabdggaHbWcbaGaeGimaadabaGaemyyae2aaSbaaWqaaiabd2eanbqabaaaniabgUIiYdGccqGGUaGlaaa@4B30@

We state our model equations, which balance the biological processes occurring in time

∂∂tp(a,t)+∂∂a((1−δ(a,t))p(a,t))=−(β(a)+μ˜(a,M(t))+ε(t))p(a,t),
 MathType@MTEF@5@5@+=feaafiart1ev1aaatCvAUfKttLearuWrP9MDH5MBPbIqV92AaeXatLxBI9gBaebbnrfifHhDYfgasaacH8akY=wiFfYdH8Gipec8Eeeu0xXdbba9frFj0=OqFfea0dXdd9vqai=hGuQ8kuc9pgc9s8qqaq=dirpe0xb9q8qiLsFr0=vr0=vr0dc8meaabaqaciaacaGaaeqabaqabeGadaaakeaadaWcaaqaaiabgkGi2cqaaiabgkGi2kabdsha0baacqWGWbaCcqGGOaakcqWGHbqycqGGSaalcqWG0baDcqGGPaqkcqGHRaWkdaWcaaqaaiabgkGi2cqaaiabgkGi2kabdggaHbaacqGGOaakcqGGOaakcqaIXaqmcqGHsisliiGacqWF0oazcqGGOaakcqWGHbqycqGGSaalcqWG0baDcqGGPaqkcqGGPaqkcqWGWbaCcqGGOaakcqWGHbqycqGGSaalcqWG0baDcqGGPaqkcqGGPaqkcqGH9aqpcqGHsislcqGGOaakcqWFYoGycqGGOaakcqWGHbqycqGGPaqkcqGHRaWkcuWF8oqBgaacaiabcIcaOiabdggaHjabcYcaSiabd2eanjabcIcaOiabdsha0jabcMcaPiabcMcaPiabgUcaRiab=v7aLjabcIcaOiabdsha0jabcMcaPiabcMcaPiabdchaWjabcIcaOiabdggaHjabcYcaSiabdsha0jabcMcaPiabcYcaSaaa@6FEC@

∂∂tn(a,t)=μ˜(a,M(t))p(a,t)−ε(t)n(a,t),
 MathType@MTEF@5@5@+=feaafiart1ev1aaatCvAUfKttLearuWrP9MDH5MBPbIqV92AaeXatLxBI9gBaebbnrfifHhDYfgasaacH8akY=wiFfYdH8Gipec8Eeeu0xXdbba9frFj0=OqFfea0dXdd9vqai=hGuQ8kuc9pgc9s8qqaq=dirpe0xb9q8qiLsFr0=vr0=vr0dc8meaabaqaciaacaGaaeqabaqabeGadaaakeaadaWcaaqaaiabgkGi2cqaaiabgkGi2kabdsha0baacqWGUbGBcqGGOaakcqWGHbqycqGGSaalcqWG0baDcqGGPaqkcqGH9aqpiiGacuWF8oqBgaacaiabcIcaOiabdggaHjabcYcaSiabd2eanjabcIcaOiabdsha0jabcMcaPiabcMcaPiabdchaWjabcIcaOiabdggaHjabcYcaSiabdsha0jabcMcaPiabgkHiTiab=v7aLjabcIcaOiabdsha0jabcMcaPiabd6gaUjabcIcaOiabdggaHjabcYcaSiabdsha0jabcMcaPiabcYcaSaaa@569D@

(1−δ(0,t))p(0,t)=2∫0aMβ(a)p(a,t)da,
 MathType@MTEF@5@5@+=feaafiart1ev1aaatCvAUfKttLearuWrP9MDH5MBPbIqV92AaeXatLxBI9gBaebbnrfifHhDYfgasaacH8akY=wiFfYdH8Gipec8Eeeu0xXdbba9frFj0=OqFfea0dXdd9vqai=hGuQ8kuc9pgc9s8qqaq=dirpe0xb9q8qiLsFr0=vr0=vr0dc8meaabaqaciaacaGaaeqabaqabeGadaaakeaacqGGOaakcqaIXaqmcqGHsisliiGacqWF0oazcqGGOaakcqaIWaamcqGGSaalcqWG0baDcqGGPaqkcqGGPaqkcqWGWbaCcqGGOaakcqaIWaamcqGGSaalcqWG0baDcqGGPaqkcqGH9aqpcqaIYaGmdaWdXaqaaiab=j7aIjabcIcaOiabdggaHjabcMcaPiabdchaWjabcIcaOiabdggaHjabcYcaSiabdsha0jabcMcaPGqaaiab+rgaKjabdggaHbWcbaGaeGimaadabaGaemyyae2aaSbaaWqaaiabd2eanbqabaaaniabgUIiYdGccqGGSaalaaa@53B6@

*p*(*a*, 0) = *p*_0_(*a*),

*n*(*a*, 0) = *n*_0_(*a*).

The left hand side of equation (2) describes the aging process for proliferating cells. On the right hand side of the same equation we find that cells of maturity *a *are lost due to three independent processes. Firstly, cells undergo mitosis, at a rate *β *depending on *a*. Such a cell shows up as two proliferating cells of maturity 0, hence the boundary condition (4). Secondly, proliferating cells are also lost due to a transition into the nonproliferating class. In contrast to proliferating cells, the nonproliferating cells do not mature and do not give rise to new cells. The rate at which the transition between the two classes occurs, μ˜
 MathType@MTEF@5@5@+=feaafiart1ev1aaatCvAUfKttLearuWrP9MDH5MBPbIqV92AaeXatLxBI9gBaebbnrfifHhDYfgasaacH8akY=wiFfYdH8Gipec8Eeeu0xXdbba9frFj0=OqFfea0dXdd9vqai=hGuQ8kuc9pgc9s8qqaq=dirpe0xb9q8qiLsFr0=vr0=vr0dc8meaabaqaciaacaGaaeqabaqabeGadaaakeaaiiGacuWF8oqBgaacaaaa@2E78@, depends on the maturity *a *of the respective cell as well as on the total number of cells *M*. We set

dadt=1−δ(a,t)
 MathType@MTEF@5@5@+=feaafiart1ev1aaatCvAUfKttLearuWrP9MDH5MBPbIqV92AaeXatLxBI9gBaebbnrfifHhDYfgasaacH8akY=wiFfYdH8Gipec8Eeeu0xXdbba9frFj0=OqFfea0dXdd9vqai=hGuQ8kuc9pgc9s8qqaq=dirpe0xb9q8qiLsFr0=vr0=vr0dc8meaabaqaciaacaGaaeqabaqabeGadaaakeaadaWcaaqaaiabdsgaKjabdggaHbqaaiabdsgaKjabdsha0baacqGH9aqpcqaIXaqmcqGHsisliiGacqWF0oazcqGGOaakcqWGHbqycqGGSaalcqWG0baDcqGGPaqkaaa@3BF7@

The function *μ*(*a*) is depicted in Figure 6B and its parameters are given in Table 1. The particular choice of a piecewise constant function is a result of the experimental observations for the control scenario (Figure 1**B**). Indeed we saw that the percentages of cells in specific phases change after day 4, as more and more cells enter the nonproliferating class. We find it plausible that a cell that has entered S-phase will finish it and therefore be less prone to entering nonproliferation. Thirdly, there is an additional time-dependent cytotoxic effect *ε *for both classes in the presence of drug. We assume the log-kill hypothesis, i.e. the cell kill is proportional to the instantaneous population [32]. Both equations are supplied with initial maturity distributions *p*_0 _and *n*_0 _at time 0.

The cytostatic action of the drug changes the maturation velocity 1 – *δ *of the proliferating cells. The ordinary differential equation

dadt=1−δ(a,t)
 MathType@MTEF@5@5@+=feaafiart1ev1aaatCvAUfKttLearuWrP9MDH5MBPbIqV92AaeXatLxBI9gBaebbnrfifHhDYfgasaacH8akY=wiFfYdH8Gipec8Eeeu0xXdbba9frFj0=OqFfea0dXdd9vqai=hGuQ8kuc9pgc9s8qqaq=dirpe0xb9q8qiLsFr0=vr0=vr0dc8meaabaqaciaacaGaaeqabaqabeGadaaakeaadaWcaaqaaiabdsgaKjabdggaHbqaaiabdsgaKjabdsha0baacqGH9aqpcqaIXaqmcqGHsisliiGacqWF0oazcqGGOaakcqWGHbqycqGGSaalcqWG0baDcqGGPaqkaaa@3BF7@

describes the characteristic curves of equation (2). Since maturation is irreversible, the function *δ *must satisfy 0 ≤ *δ *(*a*, *t*) ≤ 1. In the absence of cytostatic effects, we have *δ *= 0. Then *a *- *t *= *const*, that is cells age one-to-one with time, as stated earlier. On the other hand, if *δ *≈ 1, cell maturation is (almost) completely blocked. Observe from equation (8) that *δ *is a dimensionless quantity.

The model predicts the total cell number (1) as well as the percentages of cells in the three stages of the cell cycle. (Cells in G_2_- and M-phases are counted together.) These are defined as

G1(t)=∫0aG1(p(a,t)+n(a,t))da/M(t),S(t)=∫aG1aS(p(a,t)+n(a,t))da/M(t),G2(t)=∫aSaM(p(a,t)+n(a,t))da/M(t),
 MathType@MTEF@5@5@+=feaafiart1ev1aaatCvAUfKttLearuWrP9MDH5MBPbIqV92AaeXatLxBI9gBaebbnrfifHhDYfgasaacH8akY=wiFfYdH8Gipec8Eeeu0xXdbba9frFj0=OqFfea0dXdd9vqai=hGuQ8kuc9pgc9s8qqaq=dirpe0xb9q8qiLsFr0=vr0=vr0dc8meaabaqaciaacaGaaeqabaqabeGadaaakeaafaqadeWabaaabaGaem4raC0aaSbaaSqaaiabigdaXaqabaGccqGGOaakcqWG0baDcqGGPaqkcqGH9aqpdaWcgaqaamaapedabaGaeiikaGIaemiCaaNaeiikaGIaemyyaeMaeiilaWIaemiDaqNaeiykaKIaey4kaSIaemOBa4MaeiikaGIaemyyaeMaeiilaWIaemiDaqNaeiykaKIaeiykaKIaeeizaqMaemyyaegaleaacqaIWaamaeaacqWGHbqydaWgaaadbaGaem4raC0aaSbaaeaacqaIXaqmaeqaaaqabaaaniabgUIiYdaakeaacqWGnbqtcqGGOaakcqWG0baDcqGGPaqkaaGaeiilaWcabaGaem4uamLaeiikaGIaemiDaqNaeiykaKIaeyypa0ZaaSGbaeaadaWdXaqaaiabcIcaOiabdchaWjabcIcaOiabdggaHjabcYcaSiabdsha0jabcMcaPiabgUcaRiabd6gaUjabcIcaOiabdggaHjabcYcaSiabdsha0jabcMcaPiabcMcaPiabbsgaKjabdggaHbWcbaGaemyyae2aaSbaaWqaaiabdEeahnaaBaaabaGaeGymaedabeaaaeqaaaWcbaGaemyyae2aaSbaaWqaaiabdofatbqabaaaniabgUIiYdaakeaacqWGnbqtcqGGOaakcqWG0baDcqGGPaqkaaGaeiilaWcabaGaem4raC0aaSbaaSqaaiabikdaYaqabaGccqGGOaakcqWG0baDcqGGPaqkcqGH9aqpdaWcgaqaamaapedabaGaeiikaGIaemiCaaNaeiikaGIaemyyaeMaeiilaWIaemiDaqNaeiykaKIaey4kaSIaemOBa4MaeiikaGIaemyyaeMaeiilaWIaemiDaqNaeiykaKIaeiykaKIaeeizaqMaemyyaegaleaacqWGHbqydaWgaaadbaGaem4uamfabeaaaSqaaiabdggaHnaaBaaameaacqWGnbqtaeqaaaqdcqGHRiI8aaGcbaGaemyta0KaeiikaGIaemiDaqNaeiykaKcaaiabcYcaSaaaaaa@9D25@

where the boundaries between compartments aG1
 MathType@MTEF@5@5@+=feaafiart1ev1aaatCvAUfKttLearuWrP9MDH5MBPbIqV92AaeXatLxBI9gBaebbnrfifHhDYfgasaacH8akY=wiFfYdH8Gipec8Eeeu0xXdbba9frFj0=OqFfea0dXdd9vqai=hGuQ8kuc9pgc9s8qqaq=dirpe0xb9q8qiLsFr0=vr0=vr0dc8meaabaqaciaacaGaaeqabaqabeGadaaakeaacqWGHbqydaWgaaWcbaGaem4raC0aaSbaaWqaaiabigdaXaqabaaaleqaaaaa@3062@ and *a*_*S *_are chosen on the basis of experimental observations and *a*_*M *_is the maximum age (see Table 1 and Figure 6B). More precisely, the control scenario data were used to fix the parameters aG1
 MathType@MTEF@5@5@+=feaafiart1ev1aaatCvAUfKttLearuWrP9MDH5MBPbIqV92AaeXatLxBI9gBaebbnrfifHhDYfgasaacH8akY=wiFfYdH8Gipec8Eeeu0xXdbba9frFj0=OqFfea0dXdd9vqai=hGuQ8kuc9pgc9s8qqaq=dirpe0xb9q8qiLsFr0=vr0=vr0dc8meaabaqaciaacaGaaeqabaqabeGadaaakeaacqWGHbqydaWgaaWcbaGaem4raC0aaSbaaWqaaiabigdaXaqabaaaleqaaaaa@3062@ and *a*_*S*_. We compared the fractions of cells in each phase over time, determined by analysis of DNA content, to the model simulation from equations (9). Following [33], we have chosen the distribution of intermitotic times *φ *to be a specific shifted Γ-distribution

ϕ(a)=Φ(a−am;ρ,σ)=(a−am)e−(a−am)/σσ2.
 MathType@MTEF@5@5@+=feaafiart1ev1aaatCvAUfKttLearuWrP9MDH5MBPbIqV92AaeXatLxBI9gBaebbnrfifHhDYfgasaacH8akY=wiFfYdH8Gipec8Eeeu0xXdbba9frFj0=OqFfea0dXdd9vqai=hGuQ8kuc9pgc9s8qqaq=dirpe0xb9q8qiLsFr0=vr0=vr0dc8meaabaqaciaacaGaaeqabaqabeGadaaakeaaiiGacqWFvpGAcqGGOaakcqWGHbqycqGGPaqkcqGH9aqpcqqHMoGrcqGGOaakcqWGHbqycqGHsislcqWGHbqydaWgaaWcbaGaemyBa0gabeaakiabcUda7iab=f8aYjabcYcaSiab=n8aZjabcMcaPiabg2da9maalaaabaGaeiikaGIaemyyaeMaeyOeI0Iaemyyae2aaSbaaSqaaiabd2gaTbqabaGccqGGPaqkcqWGLbqzdaahaaWcbeqaaiabgkHiTiabcIcaOiabdggaHjabgkHiTiabdggaHnaaBaaameaacqWGTbqBaeqaaSGaeiykaKIaei4la8Iae83WdmhaaaGcbaGae83Wdm3aaWbaaSqabeaacqaIYaGmaaaaaOGaeiOla4caaa@57BB@

Here *a*_*m *_is the minimal maturity a cell has to reach before it can divide. The parameter *σ *determines the standard deviation of *ϕ *(see Table 1 and Figure 6A). The corresponding age-dependent proliferation rate is given by

β(a)=ϕ(a)α(a),
 MathType@MTEF@5@5@+=feaafiart1ev1aaatCvAUfKttLearuWrP9MDH5MBPbIqV92AaeXatLxBI9gBaebbnrfifHhDYfgasaacH8akY=wiFfYdH8Gipec8Eeeu0xXdbba9frFj0=OqFfea0dXdd9vqai=hGuQ8kuc9pgc9s8qqaq=dirpe0xb9q8qiLsFr0=vr0=vr0dc8meaabaqaciaacaGaaeqabaqabeGadaaakeaaiiGacqWFYoGycqGGOaakcqWGHbqycqGGPaqkcqGH9aqpdaWcaaqaaiab=v9aQjabcIcaOiabdggaHjabcMcaPaqaaiab=f7aHjabcIcaOiabdggaHjabcMcaPaaacqGGSaalaaa@3CA2@

where

α(a)=∫a∞ϕ(s)ds
 MathType@MTEF@5@5@+=feaafiart1ev1aaatCvAUfKttLearuWrP9MDH5MBPbIqV92AaeXatLxBI9gBaebbnrfifHhDYfgasaacH8akY=wiFfYdH8Gipec8Eeeu0xXdbba9frFj0=OqFfea0dXdd9vqai=hGuQ8kuc9pgc9s8qqaq=dirpe0xb9q8qiLsFr0=vr0=vr0dc8meaabaqaciaacaGaaeqabaqabeGadaaakeaaiiGacqWFXoqycqGGOaakcqWGHbqycqGGPaqkcqGH9aqpdaWdXaqaaiab=v9aQjabcIcaOiabdohaZjabcMcaPiabbsgaKHqaciab+nhaZbWcbaGaemyyaegabaGaeyOhIukaniabgUIiYdaaaa@3EE6@

is the fraction of cells that reach age *a *without division (see Figure 6A). We allow for a certain percentage to reach maximum age *a*_*M *_without division. As argued by [34], the mean duration of the cell cycle in solid tumors is relatively constant and therefore not influenced by the total cell number *M*.

Another source of uncertainty lies in the initial age distributions *p*_0 _and *q*_0 _at time 0. The experimental data indicated that initially proliferating cells in all stages of the cell cycle were present while there were no nonproliferating cells. It should be remarked that in the absence of nonlinear crowding effects one would observe asynchronous exponential growth. That is, the total cell number would grow exponentially with a certain well-defined rate *λ *while the percentage of cells in each age bracket would approach a steady state. This phenomenon has been widely studied in the population dynamics literature; we refer here to [35] and the references therein.

How do we let the drug act on the cells? We want to test the hypothesis that only cells in G_1_-phase are blocked while cells in other phases remain unaffected. Hence, we let

δ(a,t)=δG1(d)tT{1if 0≤a≤aG10otherwise,
 MathType@MTEF@5@5@+=feaafiart1ev1aaatCvAUfKttLearuWrP9MDH5MBPbIqV92AaeXatLxBI9gBaebbnrfifHhDYfgasaacH8akY=wiFfYdH8Gipec8Eeeu0xXdbba9frFj0=OqFfea0dXdd9vqai=hGuQ8kuc9pgc9s8qqaq=dirpe0xb9q8qiLsFr0=vr0=vr0dc8meaabaqaciaacaGaaeqabaqabeGadaaakeaaiiGacqWF0oazcqGGOaakcqWGHbqycqGGSaalcqWG0baDcqGGPaqkcqGH9aqpcqWF0oazdaWgaaWcbaGaem4raC0aaSbaaWqaaiabigdaXaqabaaaleqaaOGaeiikaGIaemizaqMaeiykaKYaaSaaaeaacqWG0baDaeaacqWGubavaaWaaiqabeaafaqabeGacaaabaGaeGymaedabaGaeeyAaKMaeeOzayMaeeiiaaIaeGimaaJaeyizImQaemyyaeMaeyizImQaemyyae2aaSbaaSqaaiabdEeahnaaBaaameaacqaIXaqmaeqaaaWcbeaaaOqaaiabicdaWaqaaiabb+gaVjabbsha0jabbIgaOjabbwgaLjabbkhaYjabbEha3jabbMgaPjabbohaZjabbwgaLjabcYcaSaaaaiaawUhaaaaa@5BA8@

where *d *is the drug concentration, δG1
 MathType@MTEF@5@5@+=feaafiart1ev1aaatCvAUfKttLearuWrP9MDH5MBPbIqV92AaeXatLxBI9gBaebbnrfifHhDYfgasaacH8akY=wiFfYdH8Gipec8Eeeu0xXdbba9frFj0=OqFfea0dXdd9vqai=hGuQ8kuc9pgc9s8qqaq=dirpe0xb9q8qiLsFr0=vr0=vr0dc8meaabaqaciaacaGaaeqabaqabeGadaaakeaaiiGacqWF0oazdaWgaaWcbaGaem4raC0aaSbaaWqaaiabigdaXaqabaaaleqaaaaa@30C3@ (*d*) ≤ 1 is a dose-dependent rate corresponding to maximum blocking effect and *T = *144 *h *is the duration of the experiment. We decided to let the action increase linearly with respect to time throughout the entire duration of the experiment. When we tried the simplest way to model time dependence, namely a sudden switch from 0 to a constant δG1
 MathType@MTEF@5@5@+=feaafiart1ev1aaatCvAUfKttLearuWrP9MDH5MBPbIqV92AaeXatLxBI9gBaebbnrfifHhDYfgasaacH8akY=wiFfYdH8Gipec8Eeeu0xXdbba9frFj0=OqFfea0dXdd9vqai=hGuQ8kuc9pgc9s8qqaq=dirpe0xb9q8qiLsFr0=vr0=vr0dc8meaabaqaciaacaGaaeqabaqabeGadaaakeaaiiGacqWF0oazdaWgaaWcbaGaem4raC0aaSbaaWqaaiabigdaXaqabaaaleqaaaaa@30C3@ > 0, the model predicted oscillations in the percentages that were not present in the experimental data. At the higher concentration of 2 *μM *it becomes necessary to introduce an action of the drug on cells in G_2_/M-phase, similar to equation (11). In order to explain the decrease of cell counts from day 5 to day 6 we assume that the additional mortality is of the type

*ε*(*t*) = max{0, *ε*_0_(*d*)·(*t *- 120)}.

We list the numerical values of the parameters that are fixed throughout all scenarios in Table 1. The values of the parameters δG1
 MathType@MTEF@5@5@+=feaafiart1ev1aaatCvAUfKttLearuWrP9MDH5MBPbIqV92AaeXatLxBI9gBaebbnrfifHhDYfgasaacH8akY=wiFfYdH8Gipec8Eeeu0xXdbba9frFj0=OqFfea0dXdd9vqai=hGuQ8kuc9pgc9s8qqaq=dirpe0xb9q8qiLsFr0=vr0=vr0dc8meaabaqaciaacaGaaeqabaqabeGadaaakeaaiiGacqWF0oazdaWgaaWcbaGaem4raC0aaSbaaWqaaiabigdaXaqabaaaleqaaaaa@30C3@ (*d*) and *ε*_0_(*d*) that differ for specific doses are stated in Table 2. We find that the dependence of δG1
 MathType@MTEF@5@5@+=feaafiart1ev1aaatCvAUfKttLearuWrP9MDH5MBPbIqV92AaeXatLxBI9gBaebbnrfifHhDYfgasaacH8akY=wiFfYdH8Gipec8Eeeu0xXdbba9frFj0=OqFfea0dXdd9vqai=hGuQ8kuc9pgc9s8qqaq=dirpe0xb9q8qiLsFr0=vr0=vr0dc8meaabaqaciaacaGaaeqabaqabeGadaaakeaaiiGacqWF0oazdaWgaaWcbaGaem4raC0aaSbaaWqaaiabigdaXaqabaaaleqaaaaa@30C3@ (*d*) on the drug dosage *d *is described very well by the equation

δG1(d)=c1d1+c1d
 MathType@MTEF@5@5@+=feaafiart1ev1aaatCvAUfKttLearuWrP9MDH5MBPbIqV92AaeXatLxBI9gBaebbnrfifHhDYfgasaacH8akY=wiFfYdH8Gipec8Eeeu0xXdbba9frFj0=OqFfea0dXdd9vqai=hGuQ8kuc9pgc9s8qqaq=dirpe0xb9q8qiLsFr0=vr0=vr0dc8meaabaqaciaacaGaaeqabaqabeGadaaakeaaiiGacqWF0oazdaWgaaWcbaGaem4raC0aaSbaaWqaaiabigdaXaqabaaaleqaaOGaeiikaGIaemizaqMaeiykaKIaeyypa0ZaaSaaaeaacqWGJbWydaWgaaWcbaGaeGymaedabeaakiabdsgaKbqaaiabigdaXiabgUcaRiabdogaJnaaBaaaleaacqaIXaqmaeqaaOGaemizaqgaaaaa@3E44@

**Table 2:** Cytostatic and cytotoxic effects as functions of drug dose. See equations (11) and (12) for explanations

drug concentration (*d*, *μM*)	0.1	0.5	1	2
δG1 MathType@MTEF@5@5@+=feaafiart1ev1aaatCvAUfKttLearuWrP9MDH5MBPbIqV92AaeXatLxBI9gBaebbnrfifHhDYfgasaacH8akY=wiFfYdH8Gipec8Eeeu0xXdbba9frFj0=OqFfea0dXdd9vqai=hGuQ8kuc9pgc9s8qqaq=dirpe0xb9q8qiLsFr0=vr0=vr0dc8meaabaqaciaacaGaaeqabaqabeGadaaakeaaiiGacqWF0oazdaWgaaWcbaGaem4raC0aaSbaaWqaaiabigdaXaqabaaaleqaaaaa@30C3@ (*d*) (dimensionless)	0.3	0.6	0.77	0.92
δG2 MathType@MTEF@5@5@+=feaafiart1ev1aaatCvAUfKttLearuWrP9MDH5MBPbIqV92AaeXatLxBI9gBaebbnrfifHhDYfgasaacH8akY=wiFfYdH8Gipec8Eeeu0xXdbba9frFj0=OqFfea0dXdd9vqai=hGuQ8kuc9pgc9s8qqaq=dirpe0xb9q8qiLsFr0=vr0=vr0dc8meaabaqaciaacaGaaeqabaqabeGadaaakeaaiiGacqWF0oazdaWgaaWcbaGaem4raC0aaSbaaWqaaiabikdaYaqabaaaleqaaaaa@30C5@ (*d*) (dimensionless)	0	0	0	0.8
*ε*_0_(*d*) (*h*^-1^)	6·10^-4^	1.1·10^-3^	1.3·10^-3^	1.8·10^-3^

with *c*_1 _= 3.5 (Figure 3**A**). A similar behavior can be surmised for the dependence of the cytotoxic effect *ε*_0_(*d*), which follows

ε0(d)=c2d1+c3d
 MathType@MTEF@5@5@+=feaafiart1ev1aaatCvAUfKttLearuWrP9MDH5MBPbIqV92AaeXatLxBI9gBaebbnrfifHhDYfgasaacH8akY=wiFfYdH8Gipec8Eeeu0xXdbba9frFj0=OqFfea0dXdd9vqai=hGuQ8kuc9pgc9s8qqaq=dirpe0xb9q8qiLsFr0=vr0=vr0dc8meaabaqaciaacaGaaeqabaqabeGadaaakeaaiiGacqWF1oqzdaWgaaWcbaGaeGimaadabeaakiabcIcaOiabdsgaKjabcMcaPiabg2da9maalaaabaGaem4yam2aaSbaaSqaaiabikdaYaqabaGccqWGKbazaeaacqaIXaqmcqGHRaWkcqWGJbWydaWgaaWcbaGaeG4mamdabeaakiabdsgaKbaaaaa@3CFB@

with *c*_2 _= 4.7·10^-3 ^and *c*_3 _= 2.1 (Figure 3**B**). The functional relationships (13) and (14) are usable for any dose within a certain range.

## Competing interests

The author(s) declare that they have no competing interests.

## Authors' contributions

PH and SEW jointly conceived the study. SEW carried out the experiments. PH created the mathematical model, performed the analysis of the experimental data and drafted the manuscript. CLA participated in the design and coordination of the study. GFW helped to create the mathematical model and participated in writing the manuscript. All authors read and approved the final manuscript.
